# Extending the Reach of the Inferior Trapezius Flap in Occipital Reconstruction: A Technical Refinement with Dorsal Scapular Artery Sacrifice—A Case Report

**DOI:** 10.3390/jcm15103933

**Published:** 2026-05-20

**Authors:** Ioana-Maria Onați, Florian Dorel Bodog, Iones Afana, Isabelle-Yvette Indig, Camelia Crișan, Cristina Mihaela Brisc, Iulia Codruța Macovei, Narcis Vîlceanu, Ruxandra Florina Bodog

**Affiliations:** 1 Medicine Program of Study, Faculty of Medicine and Pharmacy, University of Oradea, 410073 Oradea, Romania; onati.ioanamaria@student.uoradea.ro (I.-M.O.); indig.isabelleyvette@student.uoradea.ro (I.-Y.I.); crisan.camelia@student.uoradea.ro (C.C.); 2 Doctoral School of Biomedical Sciences, University of Oradea, 410087 Oradea, Romania; fbodog@uoradea.ro (F.D.B.); brisccristina@uoradea.ro (C.M.B.); 3 Plastic and Reconstructive Surgery Department, Bihor Emergency Clinical County Hospital, 410169 Oradea, Romania; younas_afana@yahoo.com; 4 Department of Surgical Disciplines, Faculty of Medicine and Pharmacy, University of Oradea, 410073 Oradea, Romania; 5 Department of Medical Disciplines, Faculty of Medicine and Pharmacy, University of Oradea, 410073 Oradea, Romania; 6 Department of Surgery, Faculty of Medicine and Pharmacy University of Oradea, 410073 Oradea, Romania; cmacovei@uoradea.ro; 7 Pelican Hospital, 410450 Oradea, Romania; 8 Department of Morphological Disciplines, Faculty of Medicine and Pharmacy, University of Oradea, 410073 Oradea, Romania

**Keywords:** occipital defect, dura mater exposure, trapezius flap, transverse cervical artery, occipital reconstruction, pedicled flap, regional flap, moderate size defect

## Abstract

**Background/Objectives**: Occipital defects with dural exposure represent a complex reconstructive challenge requiring reliable vascularized coverage to prevent severe complications. This study aims to describe a salvage reconstructive approach using a transverse cervical artery (TCA)-based inferior trapezius flap and to highlight a technical modification intended to increase flap reach. **Methods**: We report the case of a 61-year-old male presenting with a chronic occipital defect associated with infection following cerebellar abscess evacuation. After failure of primary closure, skin grafting, and local flap reconstruction, a regional pedicled inferior trapezius musculocutaneous flap was performed. Intraoperatively, the dorsal scapular artery (DSA) was selectively sacrificed after confirmation of dominant TCA perfusion to increase the arc of rotation. Flap perfusion was assessed clinically. **Results**: The flap provided adequate coverage of the defect with stable perfusion. The postoperative course was favorable, with resolution of a minor donor-site seroma. At 1- and 3-month follow-up, stable coverage, preserved cervical mobility, and satisfactory aesthetic outcomes were observed. These results were maintained at 1-year follow-up, with no functional limitation or late complications. **Conclusions**: This single case report suggests that a TCA-based inferior trapezius flap may represent a feasible salvage option in selected occipital defects. The intentional sacrifice of the DSA appeared to increase flap reach in this case; however, its safety and reproducibility remain uncertain. Further studies are required before this approach can be routinely recommended.

## 1. Introduction

Occipital bone defects with dural exposure represent a rare but clinically significant and potentially life-threatening condition. Their etiology is heterogeneous, encompassing congenital malformations—particularly encephaloceles—as well as trauma, infections, primary or metastatic tumors, and complications following neurosurgical procedures such as decompressive craniectomy. Additional factors such as fibrous dysplasia and adjuvant oncologic therapies (radiotherapy and chemotherapy) may further impair bone integrity and tissue healing [1,2,3].

Neoplastic processes in this region may remain clinically silent for prolonged periods, often resulting in extensive soft tissue and osseous destruction prior to diagnosis. Surgical resection of such lesions frequently leaves the dura mater—or even underlying neural structures—unprotected, a situation also encountered after aggressive debridement of infected or necrotic tissue [3,4].

Failure to achieve timely reconstruction of cranial defects with exposed dura mater is associated with multiple severe complications, including infection, direct cerebral injury, cerebrospinal fluid (CSF) leakage, hydrocephalus, external cerebral herniation, subdural hygroma, seizure disorders, and local wound complications such as ulceration and necrosis [5]. Dural exposure further predisposes patients to serious infectious complications such as meningitis and arachnoiditis, with potential progression to sepsis and thromboembolic events [2,5,6,7].

CSF leakage represents a particularly significant complication, frequently associated with persistent intracranial hypotension and symptoms such as postural headache, nausea, photophobia, and ataxia. Severe cases may lead to pseudomeningocele formation, nerve root entrapment, intracranial hemorrhage, and fistula formation, increasing the risk of recurrent infections [8].

The reconstructive management of occipital defects with dural exposure follows an algorithm-based approach depending on defect size, tissue quality, and patient-specific factors [2,9]. Primary closure and local advancement techniques, such as V-Y flaps, are generally reserved for small defects. For moderate defects, local rotational, transposition, or keystone flaps may provide adequate coverage, although their use is often limited by the intrinsic inelasticity of the scalp. Larger or more complex defects frequently require regional pedicled flaps—including trapezius, latissimus dorsi, or pectoralis major flaps—or microvascular free tissue transfer, which provides greater versatility but increases operative complexity [2,7,8,9,10,11].

Adjunctive techniques, including negative pressure wound therapy, dermal substitutes, and tissue expansion, may further enhance reconstructive outcomes in selected cases [12,13]. Regional flaps remain particularly valuable in patients with compromised local tissue or when microsurgical procedures are not feasible, offering reliable vascularized coverage with lower operative burden [2,9,11].

In this context, the present report aims to describe a multidisciplinary salvage strategy employing a transverse cervical artery (TCA)-based inferior trapezius flap for durable reconstruction of an occipital defect with dural exposure. Emphasis is placed on technical modifications that increase flap reach and optimize coverage, potentially avoiding the need for microvascular free tissue transfer. While the trapezius flap is well established, methods to safely extend its arc of rotation remain insufficiently described, particularly regarding the impact of dorsal scapular artery (DSA) sacrifice.

## 2. Case Presentation

A 61-year-old male patient with a history of surgically treated cerebellar tumor, cranio-cerebral trauma occurring six months prior to admission, and a chronic occipital wound infection initially caused by *Serratia odorifera*, previously treated with antibiotics, presented for elective evaluation and was admitted to the Neurosurgery Department for further investigation and management.

At presentation, the patient reported persistent headache, vertigo, and balance disturbances. Clinical examination revealed a median occipital wound with purulent discharge and a well-demarcated soft tissue defect measuring 2 × 1.5 cm. Neurological assessment demonstrated a vestibulocerebellar syndrome, characterized by impaired balance and gait instability, with a positive finger-to-nose test. The patient was fully conscious, cooperative, and oriented, with a Glasgow Coma Scale (GCS) score of 15.

During hospitalization, microbiological analysis of wound secretions identified a secondary infection with methicillin-sensitive *Staphylococcus aureus* (MSSA), most likely representing superinfection following prior antibiotic therapy. Targeted antibiotic treatment was initiated based on the antibiogram, resulting in favorable clinical evolution and control of the infectious process.

After multidisciplinary evaluation and repeated plastic surgery consultations, the patient provided written informed consent for surgical intervention following detailed discussion of the diagnosis, therapeutic options, and associated risks and benefits.

The patient subsequently underwent median occipital craniectomy with evacuation of a left paramedian cerebellar abscess under general anesthesia. Although the immediate postoperative course was stable, a persistent chronic wound infection contributed to the progression of the soft tissue defect.

Following transfer to the Department of Plastic, Reconstructive, and Microsurgery, a comprehensive clinical evaluation was performed. This included a detailed assessment of defect size, depth, anatomical location, involvement of underlying structures, and condition of surrounding tissues. Particular attention was given to prior surgical interventions and infection history, given their known impact on reconstructive outcomes, as well as to the patient’s overall clinical status and comorbidities.

**Figure 1:**
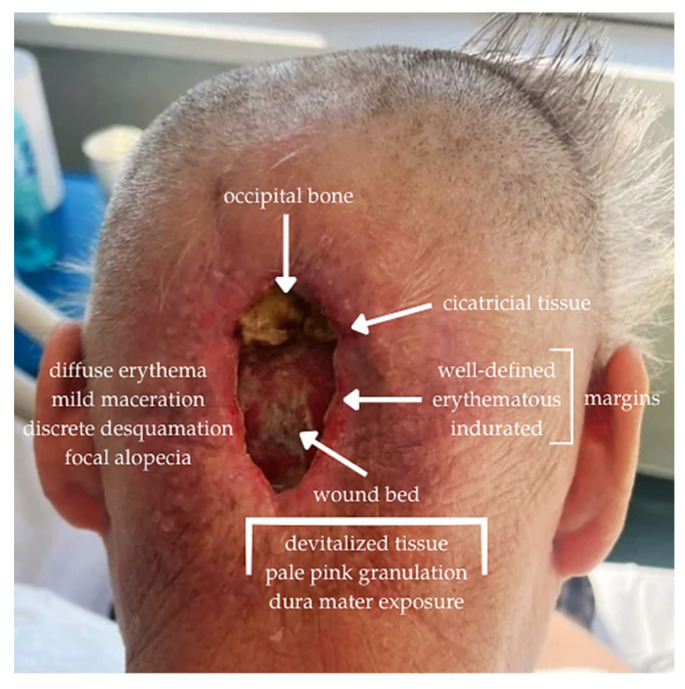
Initial clinical appearance of the occipital defect with exposed dura mater at admission. Image courtesy of Dr. Iones Afana, used with permission.

Local examination revealed a full-thickness occipital scalp defect associated with the absence of the underlying bone following a previous craniotomy, resulting in direct exposure of the dura mater. The defect was elliptical, craniocaudally oriented, and progressively enlarged to 6 × 4 × 1.5 cm. The wound margins were well-defined, everted, erythematous, and indurated, with surrounding cicatricial tissue due to repeated prior closure attempts. The wound bed demonstrated heterogeneous characteristics, including areas of devitalized tissue interspersed with pale granulation tissue. No exudate was present, and subsequent cultures were sterile following completion of antibiotic therapy. The perilesional skin showed diffuse erythema, maceration, mild desquamation, and focal alopecia, suggestive of chronic local tissue compromise and impaired vascularity (Figure 1).

**Figure 2:**
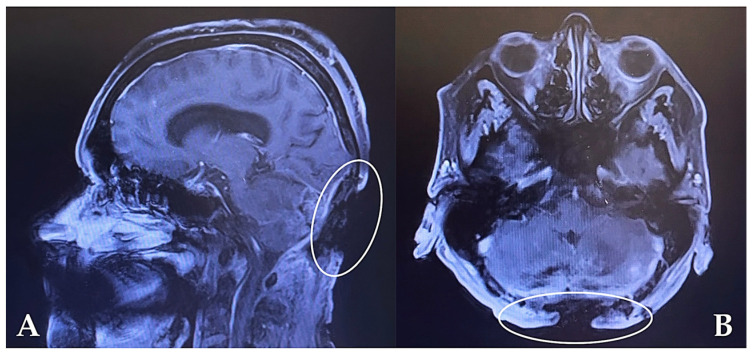
Contrast-enhanced MRI of the occipital region. (**A**) Sagittal view showing the occipital bone defect with dural involvement. (**B**) Axial view demonstrating the extent of the defect. Image courtesy of Dr. Iones Afana, used with permission.

Following completion of clinical and imaging investigations (Figure 2), a multidisciplinary approach was adopted, involving close collaboration between neurosurgical and reconstructive teams. The primary objectives included achieving stable soft-tissue coverage of the exposed dura mater, minimizing the risk of infection, preserving vascular supply, and optimizing both functional and aesthetic outcomes. Additionally, patient-specific factors such as nutritional status and local tissue perfusion were considered essential in planning the reconstructive strategy, given their impact on wound healing and postoperative outcomes.

The available options for covering the defect include primary closure with hair-bearing tissue, skin grafting, local flaps, regional flaps, and free flaps.

Primary closure was initially attempted but proved unsuccessful because of the limited elasticity of the scalp, resulting in excessive tension and wound dehiscence. Skin grafting was also unsuccessful due to the absence of a well-vascularized recipient bed. In addition, repeated surgical manipulation and local tissue compromise impaired revascularization and graft integration. Local flaps were subsequently attempted. However, their failure may be attributed to the limited mobility of the scalp, previous surgical intervention, and compromised vascularity.

Given these limitations, escalation along the reconstructive ladder was required, and a regional pedicled flap was selected based on defect size, prior reconstructive failure, and the need to avoid microsurgical free tissue transfer due to patient-specific risk factors. In addition, the presence of a previously infected and reoperated surgical field, together with the moderate defect size and the need for a shorter operative time, further supported the decision to avoid microsurgical free tissue transfer. A transverse cervical artery (TCA)-based inferior trapezius musculocutaneous flap was chosen due to its reliable vascular supply, sufficient tissue bulk, and wide arc of rotation, allowing adequate coverage of the occipital defect while avoiding the complexity of microsurgical reconstruction.

**Figure 3:**
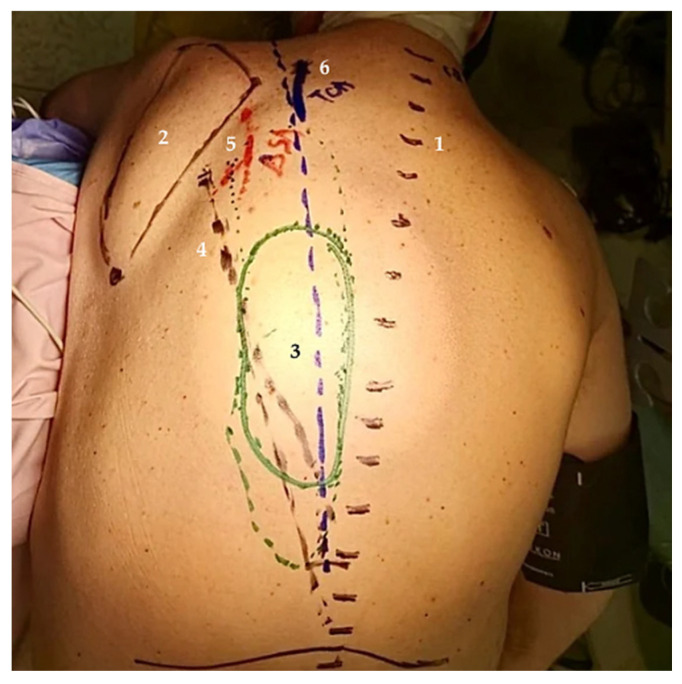
Preoperative anatomical markings of the donor site for a TCA-based inferior trapezius flap used to cover an occipital bone defect with dural exposure. (**1**) Vertebral midline. (**2**) Scapula borders and angles. (**3**) Skin paddle of the flap. (**4**) Flap axis. (**5**) DSA. (**6**) TCA. Image courtesy of Dr. Iones Afana, used with permission.

All standard preoperative protocols were followed, including anesthesia assessment, antibiotic prophylaxis, and surgical site preparation. Preoperative markings were performed to define flap design, axis, and vascular pedicles, including the TCA and dorsal scapular artery (DSA), in relation to anatomical landmarks (Figure 3).

**Figure 4:**
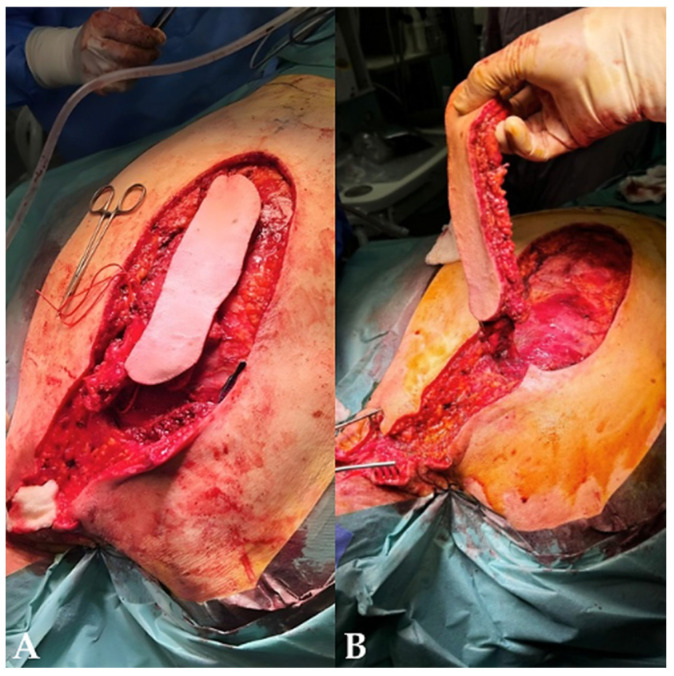
Intraoperative stages of flap elevation. (**A**) Incision and initial dissection. (**B**) Submuscular dissection and flap elevation. Image courtesy of Dr. Iones Afana, used with permission.

The surgical site was prepared and draped in a sterile manner, and the patient was positioned prone with appropriate padding and head stabilization to avoid pressure-related complications. The operative field extended craniocaudally from the occipital bone to the T12 region. Following the preoperative cutaneous markings, the incision was performed, and dissection was carried out to the submuscular plane, including the inferior part of the trapezius muscle (Figure 4).

**Figure 5:**
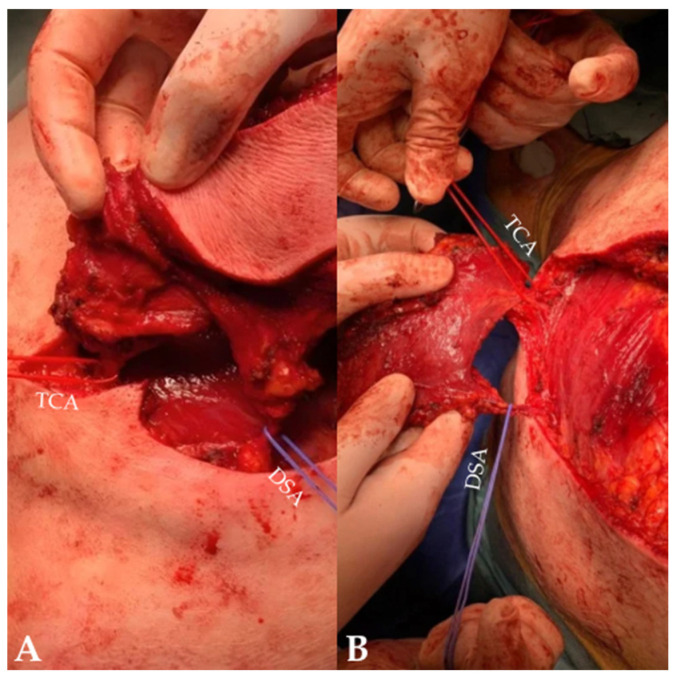
Intraoperative identification of vascular pedicles. (**A**) Identification and looping of the transverse cervical artery and dorsal scapular artery. (**B**) Flap elevation with visualization of the vascular pedicles. Image courtesy of Dr. Iones Afana, used with permission.

Both the TCA and DSA were identified (Figure 5). Careful submuscular dissection was performed along the medial border of the scapula and the deep surface of the trapezius muscle to expose and isolate both vascular pedicles. The TCA was traced laterally toward the spine, while the DSA was identified along the medial scapular border.

Both vessels were preserved during the initial stages of flap elevation to allow continuous assessment of flap perfusion. Once adequate mobilization was achieved and dominant perfusion from the TCA was confirmed, the DSA was selectively ligated to increase the arc of rotation. In cases of uncertain vascular dominance or anatomical variability, preservation of the DSA is recommended.

Flap perfusion after DSA sacrifice was assessed clinically based on capillary refill, bleeding from wound edges, and flap color. No adjunctive methods such as indocyanine green angiography or Doppler flowmetry were used. No signs of venous congestion or arterial insufficiency were observed intraoperatively. Additional perforators were ligated to enhance flap mobility.

**Figure 6:**
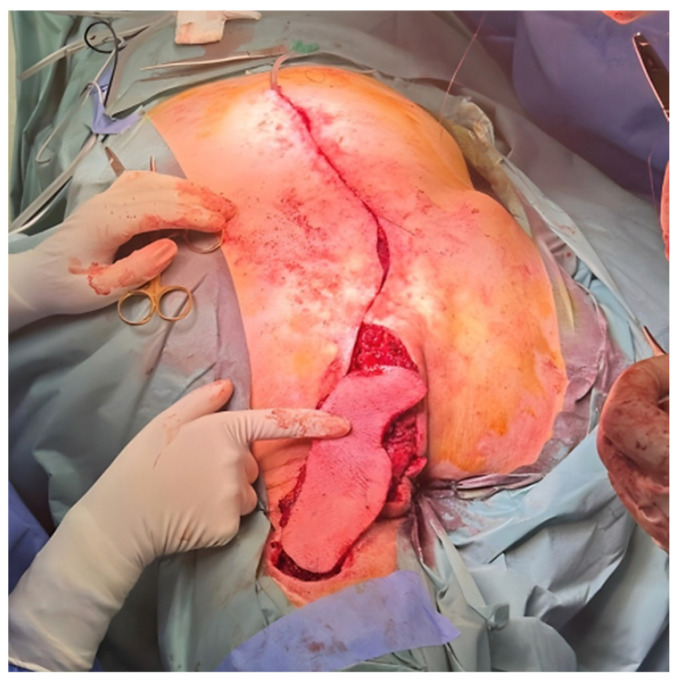
Cranial rotation and inset of the musculocutaneous flap. Image courtesy of Dr. Iones Afana, used with permission.

The musculocutaneous flap was then elevated and rotated cranially by 180°, with transposition to the defect site through a connecting incision (Figure 6). Care was taken to avoid pedicle kinking during the 180° rotation.

**Figure 7:**
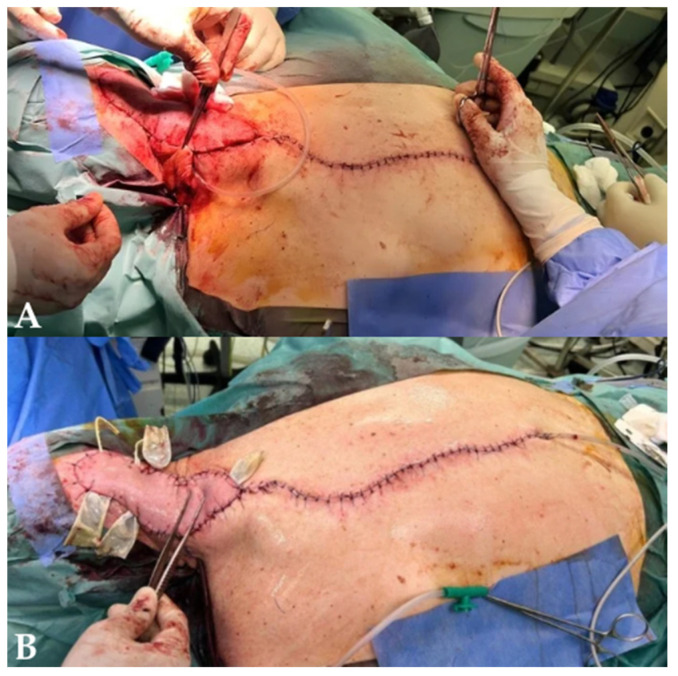
Final intraoperative result. (**A**) Flap inset at the recipient site. (**B**) Donor site closure. Image courtesy of Dr. Iones Afana, used with permission.

After thorough debridement of the recipient site to viable tissue, the flap was inset into the defect. The donor site was mobilized and closed primarily along the vertebral midline. A suction drain was placed and exteriorized laterally, and compressive dressing was applied to reduce dead space (Figure 7).

### Postoperative Outcomes

Postoperative flap perfusion remained stable throughout hospitalization, with no evidence of arterial insufficiency, venous congestion, partial flap necrosis, or wound dehiscence. Clinical assessment demonstrated adequate capillary refill, satisfactory flap colour, and preserved tissue viability during the early postoperative period.

**Figure 8:**
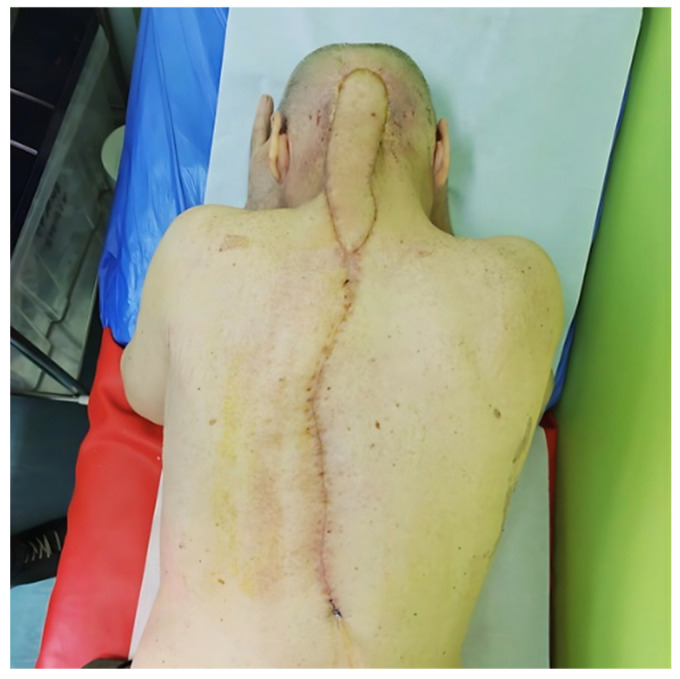
Postoperative appearance at 16 days showing favorable wound healing. Image courtesy of Dr. Iones Afana, used with permission.

The only postoperative complication consisted of a localized donor-site seroma at the inferior aspect of the surgical wound adjacent to the drainage tube. Management included maintenance of suction drainage for 14 days, ultrasound-guided aspiration on three occasions (60–80 mL each), and application of local compressive dressings to reduce dead space. Complete resolution was achieved without infection, flap compromise, or need for reoperation (Figure 8).

**Figure 9:**
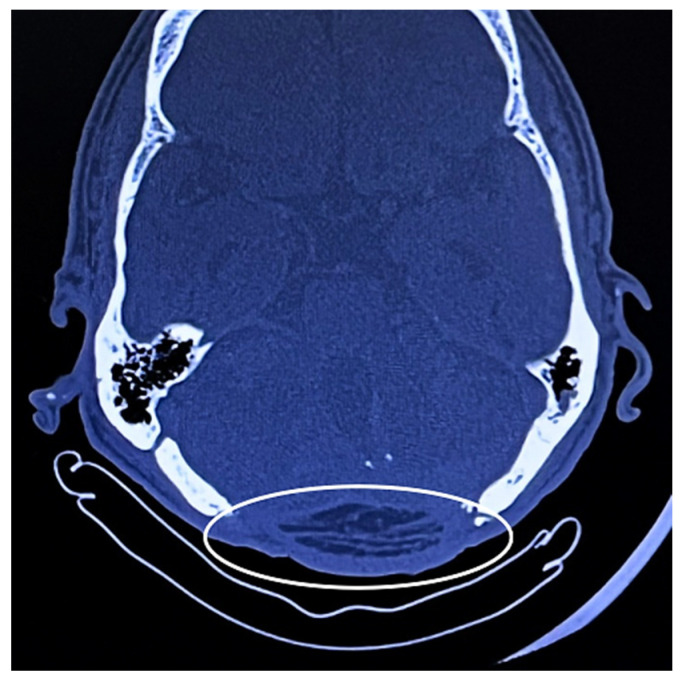
Axial cranial CT at 3 months demonstrating stable coverage of the occipital defect. Image courtesy of Dr. Iones Afana, used with permission.

At 1-month follow-up, stable soft-tissue coverage and satisfactory wound healing were observed. At 3 months, cranial CT imaging confirmed durable coverage of the occipital defect without evidence of recurrence, tissue breakdown, or fluid collection (Figure 9). Functional assessment demonstrated preserved cervical mobility, with no subjective limitation in flexion, extension, or rotation during daily activities.

At 1-year follow-up, the reconstructive result remained stable, with maintained flap viability, absence of late complications, preserved neck function, and satisfactory aesthetic outcome.

The chronological sequence of clinical events, including initial trauma, infectious complications, surgical interventions, and postoperative follow-up, is summarized in Table 1.

Clinical findings observed throughout follow-up are presented in Table 2.

This case highlights the successful salvage reconstruction of a complex occipital defect with dural exposure following failure of local techniques, using a TCA-based inferior trapezius flap. The deliberate sacrifice of the DSA to increase flap mobility represents a key technical modification that allowed adequate defect coverage in this case while maintaining favorable functional and aesthetic outcomes. This report illustrates a potential regional alternative in a high-risk reconstructive setting and may assist in surgical decision-making in situations where local options are exhausted, and microvascular free tissue transfer is not ideal or readily available.

Overall, the procedure resulted in stable soft-tissue coverage, preserved cervical mobility, and absence of major complications at 1-year follow-up.

## 3. Discussion

The report illustrates the successful salvage reconstruction of a complex occipital defect with exposed dura mater using a transverse cervical artery (TCA)-based inferior trapezius musculocutaneous flap.

Reconstruction of posterior cranial defects remains challenging due to limited local tissue availability, the convex contour of the occipital region, and the increased risk of infection and wound complications when intracranial structures are exposed [2,14]. In this setting, durable coverage with well-vascularized tissue is essential.

In our patient, repeated failure of primary closure and local flap techniques necessitated escalation to a regional pedicled solution, with the trapezius flap providing adequate coverage with favorable postoperative outcome in this case.

Unlike most reported approaches, our strategy deliberately modifies the vascular anatomy by sacrificing the dorsal scapular artery (DSA) to enhance flap mobility and reach. The deliberate sacrifice of the DSA remains controversial, as vascular dominance patterns of the trapezius flap are variable. In the absence of intraoperative perfusion imaging or anatomical confirmation, this maneuver carries a potential risk of flap compromise. We would consider DSA sacrifice only in cases where: (1) TCA dominance is clearly identified, (2) additional reach is required to cover the defect, and (3) no reliable alternative reconstructive option is available/more favorable. This approach may represent a potential option in selected cases; however, its safety and reproducibility remain uncertain.

The anatomical characteristics of the occipital scalp play a critical role in reconstructive strategy. Compared with other scalp regions, the posterior scalp demonstrates reduced elasticity due to the intrinsic rigidity of its layered structure, limiting tissue mobility and the feasibility of local advancement techniques [15,16]. From a vascular perspective, although the occipital and posterior auricular arteries form a rich subdermal plexus, effective tissue mobilization remains restricted in larger defects [10,15,16]. Furthermore, the close relationship between the scalp and underlying calvarium predisposes full-thickness defects to dural exposure, necessitating reconstruction with robust vascularized coverage capable of providing both coverage and structural support [10,17]. Within this anatomical framework, the inferior trapezius musculocutaneous flap represents a suitable option due to its reliable vascular supply from the transverse cervical system and its favorable arc of rotation toward the occipital region [18].

The reliability of the trapezius musculocutaneous flap in posterior scalp reconstruction has been well documented, particularly in cases where local options are in-sufficient [11]. Zenga et al. reported complete flap survival without major complications in a series of patients undergoing lower trapezius flap reconstruction for complex cervico-occipital defects [19], while Erdal et al. demonstrated successful defect closure without flap loss in high-risk patients using a delayed trapezius flap technique [20]. Additional studies have consistently shown favorable outcomes, including high flap survival rates and low donor-site morbidity, further supporting the trapezius flap as a dependable regional reconstructive option [18]. These findings are consistent with the outcome observed in the present case, where durable coverage was achieved following failure of local reconstructive strategies.

Microsurgical free tissue transfer remains the standard approach for extensive scalp defects, offering significant reconstructive versatility, albeit with increased operative complexity and physiological burden [14]. High success rates have been reported in the literature, supporting its role as the gold standard in large defects [21]. However, regional pedicled flaps, such as the trapezius flap, represent a valuable alternative in selected patients by reducing operative time and perioperative risk while maintaining adequate vascularized coverage [2,18]. Contemporary reconstructive algorithms support the selective use of regional flaps in moderate-sized defects or in patients with increased surgical risk [15,22], as illustrated in the present case.

The versatility of the trapezius flap is further supported by its successful application in infected or compromised wound beds. Kanayama et al. reported effective reconstruction of an infected occipital bone defect using a pedicled trapezius flap, achieving complete healing without major complications [23]. The authors highlighted the stable vascular inflow and relatively straightforward dissection of this flap, characteristics that facilitate reliable healing even in suboptimal local conditions. These findings are consistent with broader anatomical and clinical analyses demonstrating the dependable perfusion and favorable arc of rotation of the trapezius flap toward the occipital region [2,18]. To our knowledge, the deliberate sacrifice of the DSA to increase flap reach in occipital reconstruction has been only rarely described, and its practical implications remain underreported.

**Figure 10:**
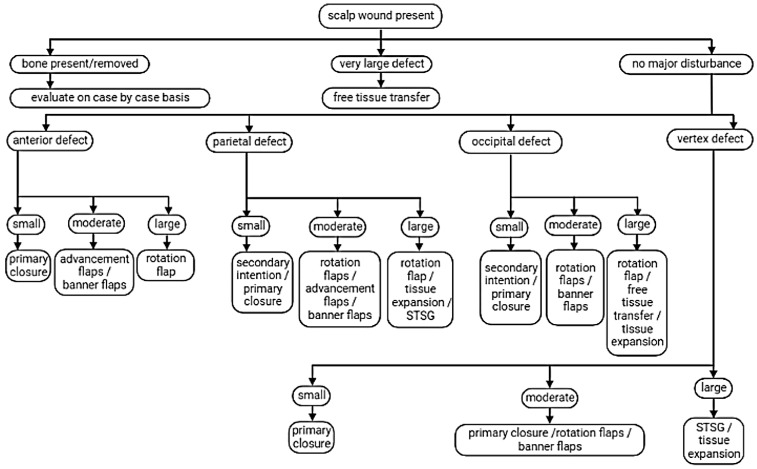
Algorithmic approach to scalp reconstruction based on defect size and location. Reproduced from [9].

From an algorithmic perspective, defect size remains a key determinant of reconstructive strategy. Large defects typically require microsurgical free tissue transfer to achieve adequate coverage and volume [16,22], whereas regional flaps may offer a more favorable balance between surgical morbidity and reconstructive reliability in moderate-sized defects. The trapezius flap, in particular, provides sufficient tissue bulk, robust vascularity, and shorter operative time compared with microsurgical alternatives, making it especially advantageous following failure of local reconstructive options [10]. In addition, local flaps such as rotational or keystone designs may be suitable for smaller defects but are often limited by scalp inelasticity and restricted donor-site laxity in the occipital region [22,24]. In the present case, the defect size and prior re-constructive failures justified the use of a regional pedicled flap, consistent with current reconstructive principles [2,22]. A summary of reconstructive options based on defect size and location, adapted from Mahmood and Eisen, is presented in Figure 10.

An important aspect highlighted by this case is the timing of escalation along the reconstructive ladder. While primary closure and local flaps remain appropriate initial strategies, prolonged reliance on insufficient techniques may delay definitive coverage and increase the risk of infection and wound chronicity. Current reconstructive algorithms emphasize early transition to well-vascularized regional or free tissue transfer when local options prove inadequate [10,22,25]. Posterior scalp defects, particularly those involving dural exposure, demonstrate limited tolerance for repeated local attempts due to reduced tissue elasticity and vascular compromise [2]. In the present case, timely escalation to a trapezius flap enabled definitive coverage and favorable healing, supporting the concept that early conversion to a reliable regional option may improve outcomes.

Despite the favorable outcome, several limitations should be acknowledged. This report describes a single case, limiting generalizability and precluding direct comparison with alternative techniques [2]. Additionally, although short- and mid-term out-comes were favorable, longer follow-up is required to fully assess long-term durability and donor-site morbidity [16]. Furthermore, while effective in moderate-sized defects, the trapezius flap may be insufficient for very large or composite defects, which may still require microsurgical reconstruction [10]. Patient-specific factors, including tissue quality, prior interventions, and comorbidities, must therefore guide individualized reconstructive decision-making [22,25]. Finally, anatomical variability of the trapezius muscle and its vascular supply should be considered, as it may influence flap reliability in selected cases [9].

From a practical perspective, this case reinforces the continued relevance of the pedicled trapezius flap in modern occipital scalp reconstruction. While free tissue transfer remains indispensable for extensive defects, regional options should not be overlooked, particularly in moderate-sized defects or in patients with increased operative risk. The trapezius flap offers reliable vascularity, adequate tissue bulk, and reduced operative time while avoiding the complexity of microsurgical reconstruction [10,18].

Notably, this report demonstrates a successful salvage application following failure of local reconstructive techniques, as well as a technical modification involving sacrifice of the dorsal scapular artery to increase the arc of rotation. In our patient, the defect measured approximately 6 × 4 cm, and definitive regional reconstruction was performed after a period of unsuccessful local management, consistent with recommended escalation strategies.

Overall, this case suggests that early escalation to regional flap reconstruction when local options fail may contribute to favorable outcomes in selected cases by avoiding repeated unsuccessful interventions and prolonged wound morbidity. Furthermore, strategic modification of flap vascular anatomy may potentially expand reconstructive options and may be considered earlier in the reconstructive algorithm in selected patients.

## 4. Conclusions

Occipital defects with dural exposure remain a complex reconstructive challenge requiring reliable vascularized coverage.

This single case report suggests that a transverse cervical artery (TCA)-based inferior trapezius flap may provide effective salvage reconstruction in selected occipital defects. The intentional sacrifice of the dorsal scapular artery (DSA) appeared to increase flap reach in this instance; however, the safety and reproducibility of this maneuver remain uncertain. Currently, there is a lack of anatomical and clinical data supporting the routine use of this maneuver.

Further anatomical studies and larger clinical series are required before this approach can be routinely recommended.

## Figures and Tables

**Table 1:** Timeline of clinical events, surgical management, and follow-up.

Timepoint	Events
6 months before admission	Cranial scalp trauma
June 2024	Chronic infected occipital wound
August 2024	Cerebellar abscess evacuation
September 2024	Repeated local closure attempts
October 2024	Regional flap reconstruction
Postoperative day 16	Favorable healing after seroma management
1 month follow-up	Stable soft-tissue coverage
1-year follow-up	Sustained functional and aesthetic outcomes without late complications

**Table 2:** Postoperative outcomes and follow-up findings.

Follow-Up	Flap status	Complications	Aesthetic
Immediate postoperative period	Stable perfusion	None	Acceptable
Postoperative day 16	Stable	Localized seroma	Satisfactory
1 month	Stable coverage	None	Satisfactory
3 months	Durable coverage on CT	None	Satisfactory
1 year	Stable result	None	Satisfactory

## Data Availability

Data is contained within the article.

## Abbreviations

The following abbreviations are used in this manuscript:

CSF	Cerebrospinal fluid
GCS	Glasgow Coma Scale
MSSA	Methicillin-sensitive Staphylococcus aureus
MRI	Magnetic Resonance Imaging
TCA	Transverse Cervical Artery
DSA	Dorsal Scapular Artery
